# Acetylated tau destabilizes the cytoskeleton in the axon initial segment and is mislocalized to the somatodendritic compartment

**DOI:** 10.1186/s13024-016-0109-0

**Published:** 2016-06-29

**Authors:** Peter Dongmin Sohn, Tara E. Tracy, Hye-In Son, Yungui Zhou, Renata E. P. Leite, Bruce L. Miller, William W. Seeley, Lea T. Grinberg, Li Gan

**Affiliations:** Gladstone Institute of Neurological Disease, University of California, San Francisco, CA 94158 USA; Neuroscience Graduate Program, University of California, San Francisco, CA 94158 USA; Department of Neurology, University of California, San Francisco, CA 94158 USA; Memory and Aging Center, Department of Neurology University of California, San Francisco, CA 94158 USA; Gladstone Institute of Virology & Immunology, San Francisco, CA 94158 USA; Physiopathology in Aging Lab/Brazilian Aging Brain Study Group—LIM22, University of Sao Paulo Medical School, Sao Paulo, Brazil

**Keywords:** Alzheimer’s disease, Neuronal polarity, Tau acetylation, Axon initial segment, Neuronal cytoskeleton

## Abstract

**Background:**

Neurons are highly polarized cells in which asymmetric axonal-dendritic distribution of proteins is crucial for neuronal function. Loss of polarized distribution of the axonal protein tau is an early sign of Alzheimer’s disease (AD) and other neurodegenerative disorders. The cytoskeletal network in the axon initial segment (AIS) forms a barrier between the axon and the somatodentritic compartment, contributing to axonal retention of tau. Although perturbation of the AIS cytoskeleton has been implicated in neurological disorders, the molecular triggers and functional consequence of AIS perturbation are incompletely understood.

**Results:**

Here we report that tau acetylation and consequent destabilization of the AIS cytoskeleton promote the somatodendritic mislocalization of tau. AIS cytoskeletal proteins, including ankyrin G and βIV-spectrin, were downregulated in AD brains and negatively correlated with an increase in tau acetylated at K274 and K281. AIS proteins were also diminished in transgenic mice expressing tauK274/281Q, a tau mutant that mimics K274 and K281 acetylation. In primary neuronal cultures, the tauK274/281Q mutant caused hyperdynamic microtubules (MTs) in the AIS, shown by live-imaging of MT mobility and fluorescence recovery after photobleaching. Using photoconvertible tau constructs, we found that axonal tauK274/281Q was missorted into the somatodendritic compartment. Stabilizing MTs with epothilone D to restore the cytoskeletal barrier in the AIS prevented tau mislocalization in primary neuronal cultures.

**Conclusions:**

Together, these findings demonstrate that tau acetylation contributes to the pathogenesis of neurodegenerative disease by compromising the cytoskeletal sorting machinery in the AIS.

**Electronic supplementary material:**

The online version of this article (doi:10.1186/s13024-016-0109-0) contains supplementary material, which is available to authorized users.

## Background

Maintenance of neuronal polarity is critical for directional information flows in neuronal networks. Loss of the polarized distribution of tau is a key early deficit in neurodegenerative diseases such as Alzheimer’s disease (AD) and frontotemporal dementia with parkinsonism linked to chromosome 17 (FTDP-17) [1–6]. In healthy neurons, tau is found predominantly in axons and at much lower levels in dendrites [7, 8]. Under pathological conditions, however, the polarized distribution of tau is disrupted, as in mouse models expressing tau with FTDP-17 mutations [9–13]. In response to cellular insults such as amyloid beta (Aβ), tau can be mislocalized to the somatodendritic compartment, leading to destruction of the cytoskeletal network in the dendrites [14, 15]. In AD mouse models, dendritic tau mediates Aβ-induced excitotoxicity by enabling transport of the kinase Fyn to dendritic spines [16, 17]. Understanding the mechanisms of the polarized distribution of tau is important. However, it is not known how the cytoskeletal sorting machinery goes awry or what triggers the mislocalization of tau to the somatodendritic compartment in a diseased neuron.

The axon initial segment (AIS), a subcellular region between the axon and the somatodendritic compartment, generates action potentials and helps maintain neuronal polarity [18]. Consisting of microtubule (MT) bundles coated with a dense submembrane protein network containing ankyrin G (AnkG), βIV-spectrin, and actin filaments [19, 20], the AIS cytoskeleton forms a barrier between the axon and the somatodendritic membrane [21] and regulates axonal entry of cargoes that require selective transport [22]. Although this barrier prevents axonal tau from invading the somatodendritic compartment, pathologically modified tau can bypass the AIS and be mislocalized from the axon [23]. The AIS cytoskeleton is perturbed in AD and other neurodegenerative diseases [24–26].

Increased acetyltransferase activity has been implicated in neurodegenerative diseases [27], and acetylation of tau increases in the early and moderate Braak stages of AD [28]. In neurodegenerative diseases with tau inclusions, acetylated tau accumulates with other pathological proteins in the soma and neurites [29, 30]. Recently, we have found that the acetylation of lysines 274 and 281 in the MT-binding domain of tau is linked to cognitive decline in human AD patients [31]. The most extensively described activity of tau is its binding to MTs [32–34], and tau regulates both static and dynamic features of neuronal MTs [35–37]. Lysine acetylation in the MT-binding domain of tau can impair its ability to stabilize MTs [38]; yet the functional significance of tau acetylation in regulating the neuronal cytoskeleton is not well understood.

In this study, we sought to determine whether AD-relevant tau acetylation affects the stability of the AIS cytoskeleton *in vivo* using transgenic mice expressing tau with mutations to mimic acetylation. To investigate how the tau-mediated disruption of AIS cytoskeleton leads to loss of axonal distribution of tau, we monitored the movement of photoconvertible tau in neuronal cultures. Finally, we assessed pharmacological stabilization of MTs as a strategy to preserve the axonal distribution of tau and reduce pathological features.

## Methods

### Plasmids

cDNA encoding 2N4R human tau was cloned into pEGFP-C1 vector (Clontech). In mApple-tagged tau plasmids, EGFP in the pEGFP-C1 vector was replaced with mApple. Tau mutations (K163/174/180/190Q, K274Q, K281Q, K274/281Q, and K274/281R) were generated with the QuickChange mutagenesis kit (Stratagene). The following plasmids were gifts: GFP-tubulin (Dr. Ron Vale, University of California, San Francisco), GFP-end-binding protein (EB) 1 (Dr. Torsten Wittmann, University of California, San Francisco), and GFP-EB3 (Dr. Niels Galjart, Erasmus MC, Rotterdam).

### Mice

The murine prion promoter (Mo.PrP) expression plasmid (Mo.PrP.Xho) has been previously described [31, 39]. Human tau WT cDNA (2N4R) or cDNA with A820C (K274Q) and A841C (K281Q) mutations were cloned into the Xho1 site of the Mo.PrP.Xho plasmid. The resulting Mo.PrP-tauWT (tauWT) and Mo.PrP-tauK274/281Q (tauKQ) transgenes were microinjected into fertilized mouse oocytes from the FVB/N genetic background and implanted into pseudopregnant female mice. The founder lines with expression of equivalent levels of tauWT and tauKQ, and higher levels of tauKQ (tauKQ^high^) in the FVB/N genetic background were then crossed with C57BL/6 mice purchased from Jackson Laboratory. All mice used for experiments were of mixed FVB/N and C57BL/6 genetic background. Tail DNA from offspring was genotyped by using the following primers: 5’ primer GGAGTTCGAAGTGATGGAAG, 3’ primer GGTTTTTGCTGGAATCCTGG. Both male and female mice were used for experiments. Mice were housed in a pathogen-free barrier facility with a 12 h light-dark cycle and ad libitum access to food and water. All animal procedures were carried out under University of California, San Francisco, Institutional Animal Care and Use Committee-approved guidelines.

### Human brain samples

Superior temporal gyrus of control and AD brains were obtained from the Mount Sinai NIH Brain and Tissue Repository (NBTR), provided by Dr. Vahram Haroutunian (The Mount Sinai School of Medicine, New York). The brain tissues were from early Braak stages 0–2 and late Braak stages 5–6, and were extracted from patients in ages of 70–103 years.

### Cell culture and transfection

HeLa cells in Dulbecco’s modified Eagle’s medium supplemented with 10 % fetal bovine serum, 100 U/ml penicillin, and 100 μg/ml streptomycin were grown at 37 °C in 5 % CO_2_. Primary cultures were established from hippocampi of Sprague-Dawley rat pups (Charles River Laboratories) on postnatal day 0 or 1. Purified cells (50,000 per 300 μl of neurobasal medium supplemented with B27; Life Technologies) were plated on poly-L-lysine-coated, glass-bottom 35-mm dishes (MatTek). After cells had attached, the medium was replaced. At 6 or 7 DIV, the cells were transfected with Lipofectamine 2000 (Life Technologies) and DNA plasmids mixed 2:1 in OPTI-MEM (Life Technologies). After 30 min, the transfection medium (neurobasal medium with kyneurenic acid) was replaced with conditioned neurobasal medium supplemented with B27.

### Immunostaining and confocal imaging

Mice were transcardially perfused with 0.9 % saline, and the brains were fixed in 4 % paraformaldehyde in PBS for 48 h and then incubated in 30 % sucrose in PBS. For antigen retrieval, coronal brain sections 30 μm thick were cut with microtome and incubated in 10 mM citric acid at 90 °C for 20 min. Floating brain sections were permeabilized and blocked with PBS containing 0.3 % Triton X-100 and 10 % normal goat serum (PBST) at room temperature for 1 h. Sections were incubated first with antibodies against AnkG (N106/36, NeuroMab) and βIV-spectrin (gift from Dr. Matthew N. Rasband, Baylor College of Medicine) at 4 °C overnight and then with Alex Fluor 488- and 555-conjugated anti-mouse and anti-rabbit antibodies (Life Technologies) at room temperature for 1 h. Immunostaining with human brains was performed as described previously [30]. Briefly, cases were selected from the Neurodegenerative Disease Brain Bank (NDBB) at the University of California, San Francisco. 8 μm-thick sections were cut from paraffin blocks and mounted on glass slides. Immunoperoxidase staining was performed using an avidin-biotin complex detection system (Vectastain ABC kit; Vector Laboratories). Slides were pretreated for antigen retrieval by immersion in 10 mM citric acid at 121 °C for 5 min. The primary antibody MAb359 [30] was incubated overnight at 4 °C and biotinylated secondary antibody (Vector Laboratories) was incubated at room temperature for 1 h. Slides were incubated in Avidin/Biotinylated enzyme Complex (ABC) at room temperature for 1 h followed by exposure to 3,3’-diaminobenzidine (DAB) substrate (Vector Laboratories). For immunofluorescence, after antigen retrieval and blocking with 0.1 % Sudan Black solution, the primary antibodies MAb359 [30], CP-13 (gift from Dr. Peter Davies, Feinstein Institute for Medical Research), and AnkG (N106/36, NeuroMab) were incubated overnight at 4 °C. The DyLight 488-conjugated anti-mouse secondary antibody was incubated at room temperature for 1 h. AIS Images were acquired with a Nikon Ti-E spinning-disk confocal microscope and a 60X oil objective. Seven serial optical sections (0.5 μm steps) were projected into a single image to visualize the AIS. ImageJ software (NIH) was used to analyze the intensity and length of the AIS.

### MT-binding assay

MT binding assays in HeLa cells were performed as described [40] with modifications. DNA plasmids of mApple-mutant tau and GFP-WT tau were co-transfected into HeLa cells. To assess the intracellular distribution of WT and mutant tau, HeLa cells at 37 °C in 5 % CO_2_ were examined with a Nikon Ti-E spinning-disk confocal microscope and a 60X oil objective 24–48 h after transfection. After conversion of fluorescent RGB images into binary images, ImageJ (NIH) was used to subtract a binary image of WT tau from that of mutant tau. Cytoplasmic tau signals after the image subtraction were calculated as the MT-unbound tau index.

### Measuring MT dynamics

Primary rat hippocampal neurons at DIV 6–7 were co-transfected with GFP-EB3 and mApple-tau; the cells were imaged at 37 °C in 5 % CO_2_ 24 h after transfection. Live time-lapse imaging was performed every second for 60 sec with a Nikon Ti-E spinning disk confocal microscope and a 60X oil objective. Movement of GFP-EB1 and GFP-EB3 comets were analyzed with the Matlab software package plusTipTracker [41].

### Fluorescence recovery after photobleaching (FRAP)

Primary rat hippocampal neurons at DIV 6–7 were co-transfected with GFP-tubulin and mApple-tau, and imaged 24 h after transfection. Before imaging, the AIS was immunolabeled with an antibody against the extracellular domain of neurofascin (A12/18, NeuroMab) at 37 °C for 5 min. After brief rinses with Neurobasal A media, Alexa Fluor 647 anti-mouse secondary antibody was applied at 37 °C for 5 min. For FRAP experiments, we used a Nikon Ti wide-field microscope and a 100X oil objective to examine cells at 37 °C in 5 % CO_2_. An ROI (~5 μm) for photobleaching was drawn in the center of the AIS, as judged from anti-neurofascin staining. GFP-tubulin was bleached with a 473-nm laser and fluorescence recovery was monitored with a 488-nm laser. Time-lapse images were taken every second for 60 sec. Images in which photobleaching reduced fluorescence intensity by more than 70 % were analyzed. The fluorescence signal was background subtracted and quantified with ImageJ (NIH).

### Photoconversion

Time-lapse live imaging during photoconversion was performed as described using photoconvertible mEOS2-tau. Primary rat hippocampal neurons at DIV 6–7 were transfected with mEOS2-tau and imaged 48–72 h later with a Nikon Ti-E spinning disk confocal microscope at 37 °C with 5 % CO_2_. A ~30-μm portion of an axon segment ~30 μm from the cell body was subjected to photoconversion with a 405-nm laser (two to three 300-ms exposures), and red fluorescence images were acquired every 30 sec for 30 min. Using ImageJ (NIH), changes in red fluorescence intensity was analyzed in both the cell body and a distal axon ~30 μm away from the photoconversion site. Changes in red fluorescence intensity were normalized to the initial red fluorescence intensity from the photoconversion site right after photoconversion.

### Western blot

Human and mouse brain tissues were lysed in RIPA buffer (50 mM Tris, pH. 7.4, 150 mM NaCl, 1 mM EDTA, 0.5 % Nonidet P-40) with histone deacetylase inhibitors (1 μM trichostatin, 5 mM nicotinamide; both from Sigma), 1 mM phenylmethyl sulfonyl fluoride (Sigma), phosphatase inhibitor cocktail (Roche), and protease inhibitor cocktail (Sigma). Lysates were sonicated and centrifuged at 170,000 *g* at 4 °C for 15 min and at 18,000 *g* at 4 °C for 10 min. Supernatants were collected, and protein concentrations were measured by bicinchoninic acid assay (Pierce). Proteins were resolved on 4–12 % SDS-PAGE and transferred to nitrocellulose membranes. After blocking with nonfat dry milk, the membranes were probed at 4 °C overnight with primary antibodies: rabbit monoclonal MAb359 for ac-K274 tau [30], rabbit monoclonal MAb63 for ac-K281 tau [31], mouse monoclonal anti-AnkG (N106/20, NeuroMab), rabbit polyclonal anti-βIV-spectrin (gift from Dr. Matthew N. Rasband, Baylor College of Medicine), mouse monoclonal Tau5 (AHB0042, Life Technologies), mouse monoclonal HT7 (MN1000, Thermo Scientific), mouse monoclonal AT8 (MN1020, Thermo Scientific), mouse monoclonal 12E8 (Prothena Biosciences), mouse monoclonal PHF-1 (gift from P. Davies), and mouse monoclonal anti-GAPDH (MAB374, Millipore). The membranes were then incubated with HRP-conjugated secondary antibodies at room temperature for 1 h. Immunoblots were visualized by enhanced chemiluminescence (Thermo Scientific) and quantified by ImageJ software (NIH).

### Statistical analyses

Data were analyzed with GraphPad Prism 5 and STATA12. Differences between groups were assessed with the unpaired *t* test, one-way ANOVA with post-hoc test, and mixed-model linear regression analysis as indicated. Longitudinal data were fitted with mixed-model linear regression using the xtmixed command from STATA 12. The linear relationship between two variables was analyzed by Pearson’s correlation analysis after natural log transformation.

## Results

### AIS cytoskeletal proteins are downregulated in human AD brains

**Fig. 1:**
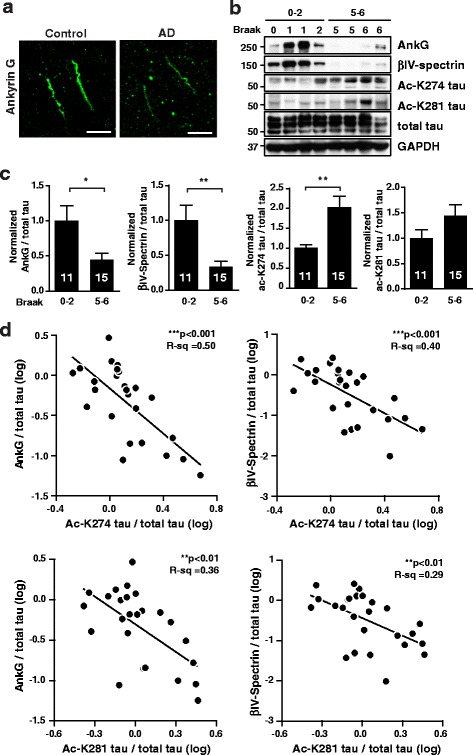
Levels of AIS cytoskeletal proteins are downregulated in human AD brains and correlate negatively with ac-K274 and ac-K281 tau levels. **a** Representative images of AnkG immunostaining in human control and AD brains. Scale bars, 10 μm. **b, c** Representative western blots and quantification of levels of AnkG, βIV-spectrin, ac-K274 tau, and ac-K281 tau in human AD brains. n = 11–15 samples/group. *p < 0.05, **p < 0.01, unpaired *t* test. **d** Correlation analyses between AnkG or βIV-spectrin and ac-K274 or ac-K281 tau. Pearson correlation analyses after natural log transformation. Values are mean ± SEM (**c**)

AIS filtering defects have been observed in primary neurons with familial AD mutations [24] and with Aβ exposure [25]. To examine the AIS in AD brain, we performed immunostaining of AnkG in the frontal cortex of AD patients. Interestingly, we observed reduced levels of AnkG in the AIS in AD brain compared to control brain (Fig. 1a). Immunoblot analyses revealed a significant decrease in levels of AIS cytoskeletal proteins, including AnkG and βIV-spectrin, in AD patients at late Braak stages compared to early Braak stages (Fig. 1b, c; Table 1). In AD brains at late Braak stages, acetyl-K274 and -K281 on tau were detected by the acetyl-lysine-specific MAb359 and MAb63 tau antibodies [31], respectively. Levels of acetyl-K274 were significantly higher at late Braak stages than early stages (Fig. 1b, c). Acetyl-K274 tau detected with MAb359 was highly enriched in intraneuronal tau inclusions in human tauopathy brains (Additional file 1: Figure S1*A-C*) [30]. Acetyl-K274 tau was localized in the cytoplasm of neurons of FTDP-17 patients (Additional file 1: Figure S1*A*) and also in the corticobasal bodies of patients with A152T mutation (Additional file 1: Figure S1*B*), which is linked with increased risk of AD and progressive supranuclear palsy (PSP) [42]. In AD patients, acetyl-K274 tau was localized in neurofibrillary tangles (Additional file 1: Figure S1*C*). Notably, the decrease in AnkG and βIV-spectrin levels in AD brains correlated with increased acetyl-K274 and -K281 on tau (Fig. 1d), raising the possibility that acetylated tau downregulates AIS cytoskeletal proteins in AD.

**Table 1:** AD human brain samples from superior temporal gyrus

Case number	Braak	Plaque load (#plaques/mm2)	Postmortemintervals (hr)
2010	0	0	18
1248	0	0	16.8
1036	0	0	4.2
1004	1	0	5
1180	1	0	28.3
1028	1	0	40.9
1279	2	0	21.7
546	2	0	4.8
1040	2	0	4.8
776	2	0	4.1
1088	2	0	14.9
625	5	0.4	7.1
399	5	9.7	3
247	5	8.4	7.3
326	5	25.2	2.9
675	5	18.4	2.2
9	5	15.6	1.8
607	6	7.6	3.9
611	6	3.04	1.5
1203	6	6.4	3
736	6	9.2	4.3
649	6	10.8	7.3
992	6	6	3.3
505	6	12	3.3
350	6	13.2	3.5
697	6	9.2	12.3

### Neuronal expression of K274/281Q tau reduces levels of AIS cytoskeleton *in vivo*

**Fig. 2:**
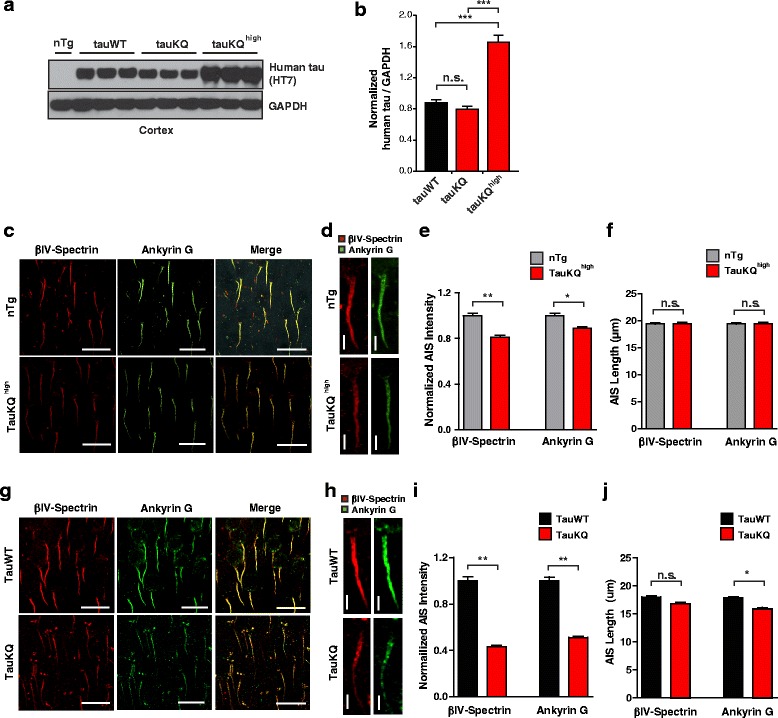
Expression of human K274/281Q tau in transgenic mice destabilizes the AIS cytoskeleton. **a, b** Levels of expression of human tau in the cortex of tauWT and tauKQ mice. n = 7-8 mice/group. ***p < 0.001, one-way ANOVA Bonferonni post-hoc analyses. **c** Representative images of βIV-spectrin and AnkG immunostaining in the somatosensory cortex of 2 month-old nontransgenic and tauKQ^high^ mice. Scale bars, 20 μm. **d-f** Representative images and quantification of the intensity and length of βIV-spectrin and AnkG immunostaining in the somatosensory cortex of 2 month-old non-transgenic and tauKQ^high^ mice. n = 119–132 cells from 6 mice/group. *p < 0.05, **p < 0.01, mixed-model linear regression analyses. Scale bars, 5 μm. **g** Representative images of βIV-spectrin and AnkG immunostaining in the somatosensory cortex of 10-12 month-old tauWT and tauKQ mice. Scale bars, 20 μm. **h-j** Representative images and quantification of intensity and length of βIV-spectrin and AnkG immunostaining in the somatosensory cortex of 10-12 month-old tauWT and tauKQ mice. n = 109-169 cells from 8 mice/group. *p < 0.05, **p < 0.01, mixed model linear regression analyses. Scale bars, 5 μm. Values are mean ± SEM

Next, we examined whether acetylated tau destabilizes the AIS cytoskeleton using transgenic mice with mouse prion promoter-driven expression of wild-type 2N4R human tau (tauWT) or tau with K274 and K281 mutated to glutamines to mimic acetylation (tauKQ) [31]. In the cortex, tauKQ and tauWT mice had comparable human tau levels, whereas tauKQ^high^ mice had significantly greater levels of tauKQ transgene expression (Fig. 2a, b). To investigate the effect of tauKQ on the AIS cytoskeleton, we analyzed βIV-spectrin and AnkG immunostaining in the somatosensory cortex of 2-month-old tauKQ^high^ mice. There was a significant decrease in βIV-spectrin and AnkG present at the AIS in tauKQ^high^ mice compared to non-transgenic controls (Fig. 2c-e), suggesting that acetylation at K274/281 can lead to destabilization of the AIS cytoskeleton *in vivo*. The length of the AIS cytoskeleton, which can be modulated by external stimuli [43, 44], did not differ in tauKQ^high^ and non-transgenic control mice (Fig. 2f). To exclude a possibility that AIS destabilization in tauKQ^high^ mice is driven by overexpression of tau transgene *per se* and is independent of acetyl-tau mimicking mutations, we further analyzed the AIS proteins in 10–12-month-old transgenic mice expressing equivalent levels of tauWT and tauKQ [31] (Fig. 2e). There was a significant reduction of both βIV-spectrin and AnkG intensity in the cortex of tauKQ mice compared to tauWT mice in 10–12 months (Fig. 2g-i). In the aged tauKQ mice, the AIS length measured by AnkG labeling was shorter than in tauWT mice (Fig. 2j), suggesting that prolonged expression of acetyl-tau mimic also compromises the integrity of the AIS besides reducing the levels of AIS proteins.

### K274/281Q tau expression increases MT dynamics in primary neurons

Acetylation at K274 and K281 in the MT-binding domain of tau occurs in human AD brains (Fig. 1b, c). Posttranslational modifications of tau in the MT-binding domain can affect its binding affinity for MTs [45–47]. To examine the effect of acetylation at K274/K281 on tau binding to MTs, we mutated K-to-Q to mimic acetyl-lysine or K-to-R to block acetylation. Substitution of lysine (K) to either glutamine (Q) or arginine (R) has been widely used in a number of studies to determine effects of acetylation of specific lysine residues on protein functions of histones, transcription factors, and enzymes [48–52]. Importantly, this lysine substitution faithfully represents the functional impact of acetylation/deacetylation on tau [31, 53, 54]. To assess the binding affinity, we analyzed cells expressing mApple-tauWT and GFP-tau mutants in a competitive MT-binding assay [40]. In HeLa cells co-transfected with mApple-tauWT and GFP-tubulin, tauWT co-localized with MTs visualized by GFP-tubulin, indicating attachment of WT tau to MTs (Additional file 1: Figure S2*A*). If a tau mutant has a lower affinity for MTs, it exhibits a more diffuse distribution than tauWT and an increased unbound tau index which is calculated by subtracting the fluorescence signal of tauWT from the tau mutant. The unbound tau index for tauK274/281Q was significantly higher than tauWT, whereas tauK274/281R showed similar binding affinity as tauWT (Additional file 1: Figure S2*B*, *C*), suggesting that acetylation at K274/281 reduces tau binding to MTs. Binding of individual mutant tauK274Q did not change, and that of tauK281Q was modestly reduced. K-to-Q mutations outside the MT-binding domain (K163/174/180/190Q; 4KQ(N)) did not affect the unbound tau index, as expected (Additional file 1: Figure S2*C*). The difference in the amount of unbound cytoplasmic tau is unlikely due to tau expression levels, as tauK274/281Q and tauWT were expressed at similar levels (Additional file 1: Figure S2*D*), and the unbound tau index did not correlate with tau levels (*p* = 0.19, Pearson correlation analysis).

Tau isoforms with different MT-binding affinities differ in their effects on the dynamic behavior of MTs [55, 56]. To determine whether acetylation at K274/281 affects MT dynamics in living cells, we tracked the movement of individual MTs by using GFP-tagged ending-binding (EB) proteins that bind to MT plus-ends [57]. The rates of movement were calculated in an unbiased fashion with plusTipTracker, which faithfully tracks EB comets from time-lapse images of living cells [41].

**Fig. 3:**
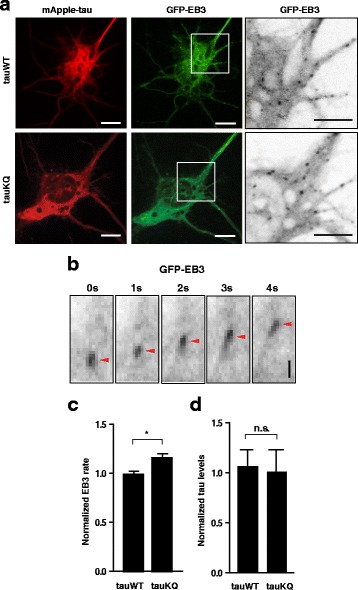
K274/281Q tau increases MT dynamics in primary neurons. **a–d** Measuring MT dynamics with GFP-EB3 in rat primary neurons. **a** Representative images of HeLa cells co-transfected with mApple-tauWT or -tauKQ and GFP-EB3. Panels on the right show enlarged images of GFP-EB3 comets within the white boxes. Scale bars, 5 μm. **b** Movement of an individual GFP-EB3 comet (arrowheads) Scale bar, 2 μm. **c, d** Quantification of GFP-EB3 movement rate and tau levels in rat primary neurons co-transfected with GFP-EB3 and mApple-tau. n = 11–13 cells/group from two independent experiments. *p < 0.05, one-way ANOVA with Tukey’s post hoc test. Values are mean ± SEM

We co-transfected primary rat hippocampal neurons at 6–7 DIV with GFP-EB3 and mApple-tau constructs. EB comets were imaged 24 to 48 h after transfection, and MT dynamics were analyzed (Fig. 3a, b). The rate of movement was significantly higher in neurons expressing tauKQ than in those expressing tauWT (Fig. 3c). The levels of tauWT and tauKQ did not differ (Fig. 3d), and the rates of movement of EB comets did not correlate with tau levels (*p* = 0.37, Pearson correlation analysis).

### K274/281Q tau expression leads to hyperdynamic MTs in the AIS

The stability of neuronal MTs varies depending on the cellular compartment. MTs in the AIS are highly stabilized by modifications and bundling and function as a barrier to maintain the polarized distribution of tau in axons [20, 23, 58]. Since the acetyl-tau mimic increased MT dynamics in neurons, we next examined the effect of tau acetylated at K274/281 on the stability of the MTs in the AIS by FRAP. We also suspected that the compromised integrity of βIV-spectrin and AnkG induced by the acetyl-tau mimic (Fig. 2c-J) could contribute to altering the stability of MTs in the AIS because submembranous cytoskeletal proteins are connected to MTs in the AIS [20, 59].

**Fig. 4:**
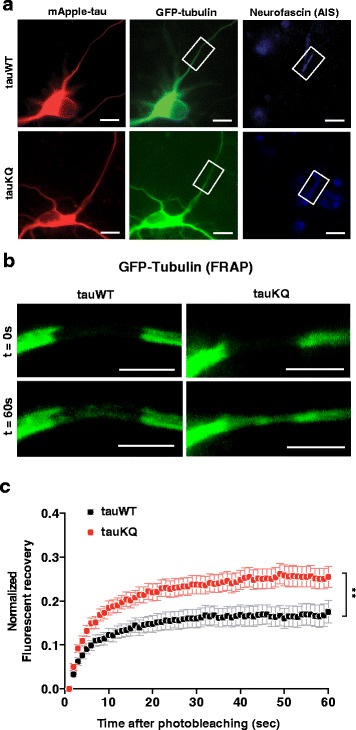
K274/281Q tau increases tubulin dynamics in the AIS. **a** Representative images of a rat primary neuron co-transfected with mApple-tauWT or -tauKQ and GFP-tubulin. White boxes indicate the portion of the AIS (revealed by anti-neurofascin) where GFP-tubulin was photobleached. **b** Representative images of photobleaching and fluorescence recovery of GFP-tubulin in a ~5-μm portion of the AIS in rat primary neurons transfected with mApple-tauWT or -tauKQ. **c** Quantification of FRAP of GFP-tubulin in the AIS of rat primary neurons co-transfected with mApple-tauWT or -tauKQ and GFP-tubulin. n = 25–26 cells/group from three independent experiments. **p < 0.01, mixed model linear regression analysis. Values are mean ± SEM. Scale bars, 5 μm

Rat hippocampal neurons at 6–7 DIV were co-transfected with GFP-tubulin and mApple-tau constructs; 24 h later, we labeled the AIS of live neurons with an antibody against the extracellular domain of neurofascin, located in the plasma membrane of the AIS [59]. The neurofascin antibody delineated the AIS during live imaging (Fig. 4a), enabling us to photobleach GFP-tubulin in a segment of the AIS. Monitoring of the GFP-tubulin signal for 1 min after photobleaching showed a significantly faster fluorescence recovery rate in cells expressing tauKQ than in those expressing similar levels of tauWT (Fig. 4b, c), consistent with destabilization of MTs in the AIS by acetylated tau.

### K274/281Q tau expression leads to the somatodendritic mislocalization of tau, which can be attenuated by EpoD

**Fig. 5:**
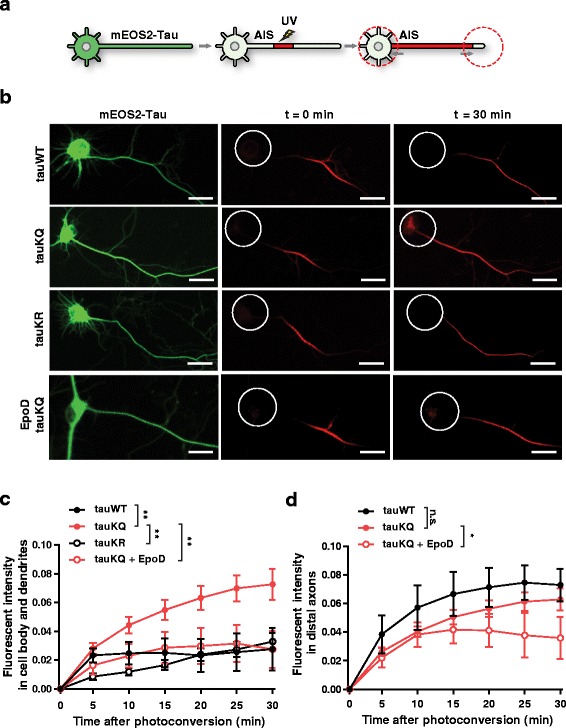
Stabilization of MTs reduces somatodendritic mislocalization of K274/281Q tau. **a–d** Photoconversion of mEOS2-tau and its movement in rat primary neurons. **a** Schematic diagram of photoconversion of mEOS2-tau in the AIS and monitoring its movement toward the somatodendritic compartment and distal axon. **b** Time-lapse live images of mEOS2-tauWT, -tauKQ, and -tauKR before and after photoconversion in an axon segment ~30 μm from the AIS. The last row represents photoconversion of mEOS2-tauKQ after EpoD treatment (20 nM). White circles indicate the somatodendritic compartment. Scale bars, 10 μm. **c, d** Quantification of fluorescent intensity in the somatodendritic compartment (**c**) and distal axon (**d**) for 30 min after photoconversion of mEOS2-tau in rat primary neurons. n = 8–21 cells/group from three to nine independent experiments. *p < 0.05, **p < 0.01, mixed model linear regression analyses. Values are mean ± SEM

Since the AIS is implicated in restricting tau distribution in the axon [23], we suspected that weakening of the AIS barrier by acetylated tau could lead to the mislocalization of axonal tau. Photoconvertible tau constructs were used to monitor the movement of tau in rat hippocampal neurons. At DIV 6–8, the cells were transfected with mEOS2-tau construct that turns red upon UV illumination. Tau was targeted for photoconversion in an AIS segment ~30 μm away from the cell body, and influx of tau into the somatodendritic compartment was monitored (Fig. 5a; Additional file 2: Movie 1, Additional file 3: Movie 2). As judged from the increase in fluorescence intensity, the influx was much greater with tauKQ than tauWT or tauK274/281R (tauKR) (Fig. 5b, c). The higher influx rate suggests that acetylation at K274/281 enabled tau to bypass the sorting barrier in the AIS. In the distal axons, cells expressing tauWT and tauKQ showed similar time-dependent increase in fluorescence intensity (Fig. 5d), suggesting that both can freely redistribute in the absence of the AIS barrier. Since hyperphosphorylation of tau can also contribute to somatodendritic mislocalization of tau [14, 15, 23, 60, 61], we analyzed the phosphorylation status of tauKQ. Acetyl-mimicking mutations at K274/281 did not alter phosphorylation at S262/S356 (12E8) and S202/T205 (AT8) in the cortex of tauKQ-expressing mice. Phosphorylation at S396/S404 (PHF1) was even significantly reduced in tauKQ mice compared to tauWT mice (Additional file 1: Figure S3*A, B*). These data support that acetylation at K274/281 induces somatodendritic mislocalization of tau by mechanisms that are independent of increased tau phosphorylation.

To test whether stabilization of MTs could restore the AIS barrier function and prevent mislocalization of acetylated tau, we treated the neurons expressing tauKQ with a low dose of epothilone D (EpoD), an MT stabilizer, to reduce MT dynamics [36, 37]. Treatment with EpoD prevented the influx of axonal tauKQ into the somatodendritic compartment (Fig. 5b, c). EpoD also modestly slowed down the movement of tauKQ to the distal portion of axon (Fig. 5d). These findings support the importance of MT stability in controlling axonal sorting of tau.

## Discussion

AIS integrity was perturbed in AD brains with increased tau acetylation. AnkG and βIV-spectrin are essential for AIS integrity since depletion of either AnkG or βIV-spectrin dismantles the AIS [62–64]. AnkG and βIV-spectrin levels were decreased in human AD brains, consistent with a report that the AIS filtering machinery was impaired in mouse and cellular models of AD [24, 25]. The level of acetylated tau increases as AD pathology proceeds [28] and, in particular, ac-K274 tau accumulates in human brains with tau inclusions (Additional file 1: Figure S1 *A-C*). In human AD brains, we found that elevated levels of both ac-K274 and ac-K281 tau were correlated negatively with the levels of AnkG and βIV-spectrin. TauKQ-expressing mice had reduced levels of the cytoskeletal proteins AnkG and βIV-spectrin in the AIS compared to non-transgenic and tauWT mice with matching transgene expression, suggesting a causative role of acetyl-K274/281 tau in AIS perturbation. This functional connection between acetyl-K274/281 tau and AIS perturbation is a novel finding that is distinct from roles of other lysine acetylation such as acetyl-K174, which slows down tau turnover and promotes its accumulation [54]. Mimicking acetyl-K274/281 did not change steady-state levels of tau in neurons (Fig. 3d), supporting the notion that these distinct effects are likely to depend on the location of the lysine residue [53]. Since the AIS cytoskeleton is critical for maintaining axonal-dendritic asymmetry [18], the downregulation of AIS cytoskeletal proteins in human and mice indicates that sorting of neuronal proteins that require polarized distribution may be impaired in AD brains with increased acetylated tau.

Pathogenic mutations increase MT turnover and perturb MT stability in transgenic mice expressing human P301S or P301L tau [36, 37]. However, little is known about the underlying mechanisms. In primary neurons, our current study showed that acetyl-mimicking K274/281Q tau led to elevated MT movements compared with cells expressing WT tau, suggesting that the acetylation of K274 and K281 promotes MT hyperdynamicity. Neurons are highly polarized cells and MT dynamics in a neuron varies depending on the subcellular location [65]. Unlike those in dendrites and distal axons, MTs in the AIS are highly stable [59, 66]. Tau acetylated at K274 and K281 reduced the stability of MTs in the AIS, as measured with site-specific FRAP to monitor tubulin dynamics. Destabilization of MTs in the AIS has been demonstrated by increased EB3 mobility in a cellular model of AD [25]. Our findings advance the understanding of the mechanism underlying AIS dysregulation by identifying key sites of tau acetylation that induces MT hyperdynamicity in the AIS. A recent study on single-molecule tracking of tau has revealed a short dynamic interaction (~40 ms) of tau with MTs [67]. Acetylation at K274/281 could change this short interaction between tau and MTs, resulting in alteration of the association/dissociation kinetics of other MT-binding molecules that control MT dynamics such as EB3.

How does acetylated tau destabilize MTs, AnkG, and βIV-spectrin in the AIS? MTs and the submembranous cytoskeleton appear to be both physically and functionally connected. MT bundles in the AIS are densely coated with an actin-based cytoskeletal network that contains AnkG and βIV-spectrin [20]. EB1 and EB3 may connect MTs to AnkG, and EB1/3 knockdown leads to downregulation of AnkG in the AIS [59]. On the other hand, a mutation in ankyrin disrupts MT organization in *C. elegans* [68]. Tau interacts with EB1 and EB3 and augments their binding to MTs [69]. One likely mechanism could be that acetyl-K274/281 tau reduces EB1/3 binding to MTs, leading to destabilization of MT and downregulation of AnkG and βIV-spectrin in the AIS. Considering tau’s potential role in connecting MTs and actin-based cytoskeletal networks [70] and its interaction with actin filament [31, 71, 72], it is also possible that, independently of EB1/3, the AIS cytoskeleton in the proximity of the membrane might be destabilized by altered direct binding of acetylated tau to submembranous cytoskeletal networks consisting of actin, AnkG, and βIV-spectrin after its detachment from MTs in the AIS. Tau can interact with proteins in the membrane of the AIS since tau is shown to be associated with the membrane when tau is hypo-phosphorylated [73, 74]. Interestingly, K274/281Q tau reduces phosphorylation of S396/S404 (Additional file 1: Figure S3*A, B*), raising a possibility of increased association of acetyl-K274/281 tau with proteins in the vicinity of the membrane. On the other hand, since tau is intrinsically disordered and has multiple binding partners, including enzymes involved in signaling pathways [35, 75], acetyl-K274/281 tau can exert an indirect effect on AIS destabilization by disturbing signal transduction. For instance, tau can bind to histone deacetylase-6 (HDAC6) and inhibit the enzymatic activity of HDAC6, which may regulate MT stability via tubulin acetylation [76].

Does tau acetylation alter other posttranslational modifications of tau such as phosphorylation? Somatodendritic mislocalization of tau also depends on the phosphorylation state of tau [60, 61]. Axonal tau crosses the AIS barrier and is mislocalized to the somodendritic compartment after pharmacological treatment that increases tau phosphorylation [23]. Pseudophosphorylation at KXGS motifs of tau is sufficient to induce its somatodendritic mislocalization [23]. The effect of acetylation on the phosphorylation status of tau is lysine-specific: Acetylation at lysines of KXGS motifs (K259/K353) blocks phosphorylation at the adjacent serines in the KXGS motifs (S262/S356) [53] whereas acetylation at K174 does not alter phosphorylation at S262/S356 but increases phosphorylation at S202/T205 [54]. We observed that acetylation-mimicking mutations at K274/281 did not change phosphorylation at S262/S356 in the KXGS motifs (12E8) as well as S202/T205 (AT8) in the cortex of tauKQ mice whereas phosphorylation at S396/S404 (PHF1) was decreased in tauKQ mice compared to tauWT mice (Additional file 1: Figure S3*A, B*). These findings demonstrate that acetylation at K274/281 plays a pivotal role, not secondary to tau phosphorylation, in somatodendritic mislocalization.

Our finding that MTs in the AIS are critical for axonal retention of tau is consistent with the notion that they form a retrograde barrier that keeps axonal tau from entering the somatodendritic compartment [23]. Acetylated tau destabilizes this MT-based barrier in the AIS and thus could enter the somatodendritic compartment. MT stabilization with EpoD restored this barrier function and prevented mislocalization of acetylated tau. Pharmacological MT stabilization reduces tau binding to MTs, and EpoD dissociates tau from MTs [77, 78]. Increased diffusion of MT-free tau could contribute to circumvention of the MT-based barrier in the AIS [23]. However, low-dose EpoD prevented acetylated tau from mislocalization – a finding that emphasizes the importance of MT stability in the barrier function for retention of axonal tau. Since the submembrane AIS cytoskeleton functions as a filter controlling cytoplasmic transport in a neuron [22], reduction of AnkG and βIV-spectrin levels by acetyl-K274/281 tau might also contribute to tau mislocalization. MT stabilization with EpoD prevented tau mislocalization and thus might increase the stability of interconnected AIS submembrane cytoskeletal networks. Our findings suggest that acetylated tau has an active role in disturbing stability of MTs in the AIS, resulting in circumvention of MT-based barrier in the AIS and consequent loss of polarized localization.

## Conclusions

This study shows that AD-relevant acetylation of tau at K274 and K281 has a critical role in destabilization of AIS cytoskeleton concomitant with loss of polarized distribution of tau. AIS-specific cytoskeletal proteins were downregulated in the brains of both human AD patients and transgenic mice expressing an acetyl-tau mimic. The mutant tau that mimics acetylation (K274/281Q) had reduced affinity for MTs and increased MT dynamics in the AIS. Destabilization of the AIS cytoskeleton resulted in mislocalization of K274/281Q tau into the somatodendritic compartment. Pharmacological stabilization of MTs prevented tau mislocalization. Our findings support aberrant tau acetylation as a novel mechanism by which neuronal polarity is compromised in the pathogenesis of neurodegenerative diseases.

## Abbreviations

AD, Alzheimer’s disease; AIS, axon initial segment; DIV, days in vitro; EB, end-binding proteins; EpoD, epothilone D; FRAP, fluorescence recovery after photobleaching; MT, microtubule; PSP, progressive supranuclear palsy

## Additional files

Additional file 1: Figure S1. Ac-K274 tau inclusions in human tauopathy brains. (A-C) Representative images of ac-K274 tau immunostaining in the inferior temporal cortex of human tauopathy brains. Scale bars, 50 μm. (A) Cytoplasmic tau inclusions (arrows) in the brain of an FTDP-17 patient. (B) Corticobasal bodies (arrows) in a patient with the A152T tau mutation. (C) Neurofibrillary tangles (arrows) in an AD brain. Scale bars, 50 μm. **Figure S2**. K274/281Q tau has reduced affinity for MTs. (A) Representative images of co-localization of GFP-tubulin and mApple-tauWT in a HeLa cell. (B–D) MT-binding assay in HeLa cells co-transfected with GFP-tau mutant and mApple-tauWT. (B) Representative images of HeLa cells co-transfected with GFP-tauK274/281Q or -tauK274/281R and mApple-tauWT. GFP-tauK274/281Q is distributed diffusely in the cytoplasm. mApple-tauWT appears to bind to MTs. (C,D) Quantification of MT-unbound tau and levels of tau in HeLa cells co-transfected with GFP-mutant tau and mApple-tauWT. 4KQ(N) denotes GFP-tauK163/174/180/190Q. n = 60–90 cells/group from two to three independent experiments. ****p* < 0.001, one-way ANOVA with Dunnett’s post-hoc analyses. Values are mean ± SEM Scale bars, 10 μm. **Figure S3**. Levels of phosphorylated tau in tauWT and tauKQ mice. (A,B) Representative western blots and quantification of levels of phosphorylated human tau in the cortex of 12-13 month-old tauWT and tauKQ mice. The antibodies 12E8, AT8, and PHF1 recognize phosphorylated S262/S356, S202/T205, and S396/S404, respectively, on human tau. n = 8 mice/group. ****p* < 0.001, unpaired *t* test. Values are mean ± SEM (C). (PDF 6517 kb) Additional file 2: Movie 1. mEOS2-tauWT movement in a rat primary neuron. Time-lapse images of red mEOS2-tauWT were taken every 30 sec for 25 min after photoconversion. Location of the cell body is indicated by the dashed circle. Scale bar, 10 μm. (MP4 25 kb) Additional file 3: Movie 2. mEOS2-tauK274/281Q movement in a rat primary neuron. Time-lapse images of red mEOS2-tauKQ were taken every 30 sec for 25 min after photoconversion. Location of the cell body is indicated by the dashed circle. Scale bar, 10 μm. (MP4 44 kb)
