# Supplementary material for: Acetylated tau destabilizes the cytoskeleton in the axon initial segment and is mislocalized to the somatodendritic compartment
Source: Mol Neurodegener. 2016 Jun 29;11:47. doi: 10.1186/s13024-016-0109-0 (PMC4928318; doi:10.1186/s13024-016-0109-0)
Supplement: Additional file 1: Figure S1. — Ac-K274 tau inclusions in human tauopathy brains. (A-C) Representative images of ac-K274 tau immunostaining in the inferior temporal cortex of human tauopathy brains. Scale bars, 50 μm. (A) Cytoplasmic tau inclusions (arrows) in the brain of an FTDP-17 patient. (B) Corticobasal bodies (arrows) in a patient with the A152T tau mutation. (C) Neurofibrillary tangles (arrows) in an AD brain. Scale bars, 50 μm. Figure S2. K274/281Q tau has reduced affinity for MTs. (A) Representative images of co-localization of GFP-tubulin and mApple-tauWT in a HeLa cell. (B–D) MT-binding assay in HeLa cells co-transfected with GFP-tau mutant and mApple-tauWT. (B) Representative images of HeLa cells co-transfected with GFP-tauK274/281Q or -tauK274/281R and mApple-tauWT. GFP-tauK274/281Q is distributed diffusely in the cytoplasm. mApple-tauWT appears to bind to MTs. (C,D) Quantification of MT-unbound tau and levels of tau in HeLa cells co-transfected with GFP-mutant tau and mApple-tauWT. 4KQ(N) denotes GFP-tauK163/174/180/190Q. n = 60–90 cells/group from two to three independent experiments. ***p < 0.001, one-way ANOVA with Dunnett’s post-hoc analyses. Values are mean ± SEM Scale bars, 10 μm. Figure S3. Levels of phosphorylated tau in tauWT and tauKQ mice. (A,B) Representative western blots and quantification of levels of phosphorylated human tau in the cortex of 12-13 month-old tauWT and tauKQ mice. The antibodies 12E8, AT8, and PHF1 recognize phosphorylated S262/S356, S202/T205, and S396/S404, respectively, on human tau. n = 8 mice/group. ***p < 0.001, unpaired t test. Values are mean ± SEM (C). (PDF 6517 kb) [file 13024_2016_109_MOESM1_ESM.pdf]

Table S1

AD human brain samples from superior temporal gyrus

Case number	Braak	Plaque load (#plaques/mm2)	Postmortem intervals (hr)
2010	0	0	18
1248	0	0	16.8
1036	0	0	4.2
1004	1	0	5
1180	1	0	28.3
1028	1	0	40.9
1279	2	0	21.7
546	2	0	4.8
1040	2	0	4.8
776	2	0	4.1
1088	2	0	14.9
625	5	0.4	7.1
399	5	9.7	3
247	5	8.4	7.3
326	5	25.2	2.9
675	5	18.4	2.2
9	5	15.6	1.8
607	6	7.6	3.9
611	6	3.04	1.5
1203	6	6.4	3
736	6	9.2	4.3
649	6	10.8	7.3
992	6	6	3.3
505	6	12	3.3
350	6	13.2	3.5
697	6	9.2	12.3

Figure S1

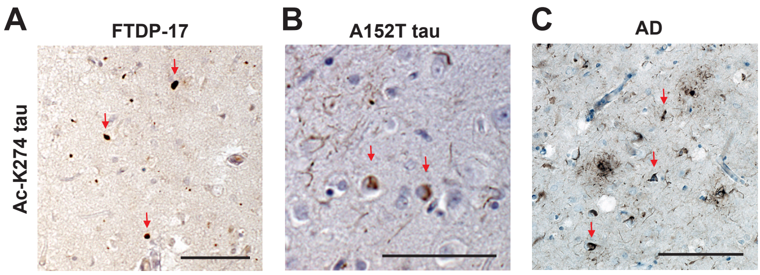

Figure S2

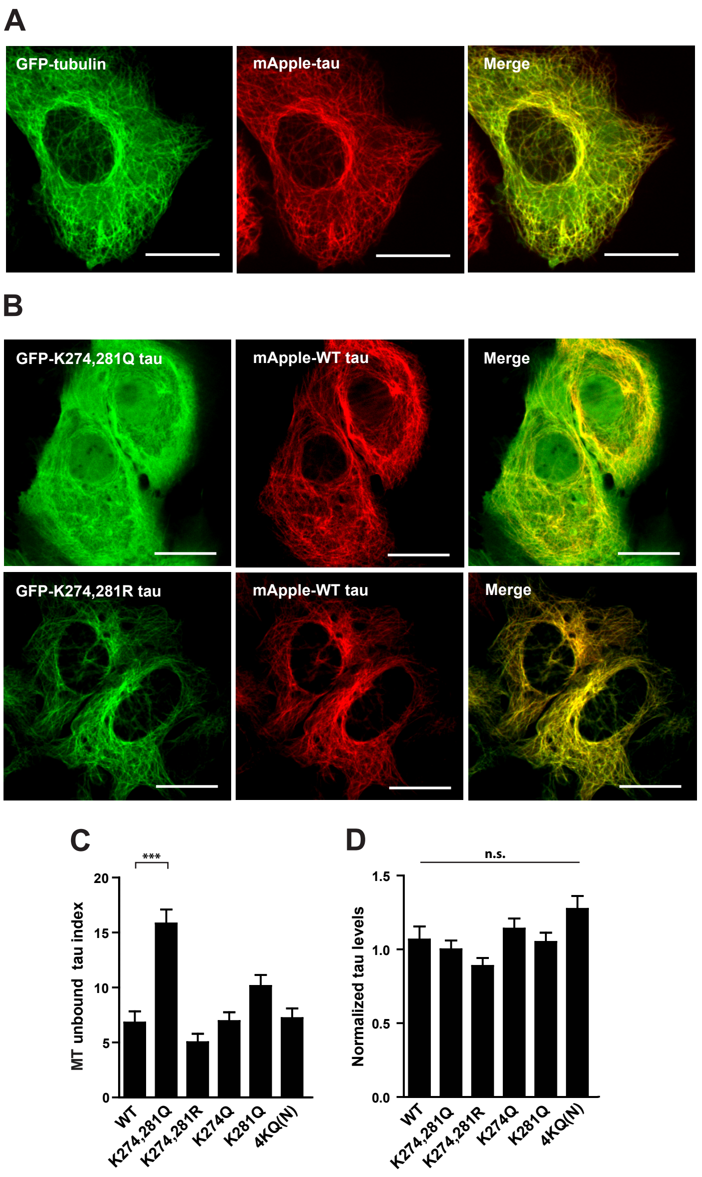
A
GFP-tubulin
mApple-tau
Merge
B
GFP-K274,281Q tau
mApple-WT tau
Merge
GFP-K274,281R tau
mApple-WT tau
Merge
C
***
MT unbound tau index
20
15
10
5
0
WT
K274,281Q
K274,281R
K274Q
K281Q
4KQ(N)
D
n.s.
Normalized tau levels
1.5
1.0
0.5
0.0
WT
K274,281Q
K274,281R
K274Q
K281Q
4KQ(N)

Figure S3

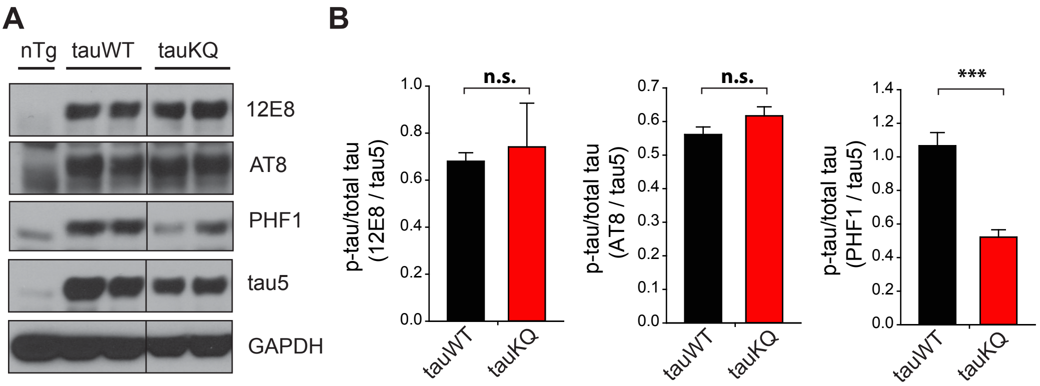
A
nTg
tauWT
tauKQ
12E8
AT8
PHF1
tau5
GAPDH
B
n.s.
p-tau/total tau (12E8 / tau5)
1.0
0.8
0.6
0.4
0.2
0.0
tauWT
tauKQ
n.s.
p-tau/total tau (AT8 / tau5)
0.7
0.6
0.5
0.4
0.3
0.2
0.1
0.0
tauWT
tauKQ
***
p-tau/total tau (PHF1 / tau5)
1.4
1.2
1.0
0.8
0.6
0.4
0.2
0.0
tauWT
tauKQ
